# Battle, Journey, Imprisonment and Burden: patterns of metaphor use in blogs about living with advanced cancer

**DOI:** 10.1186/s12904-020-00557-6

**Published:** 2020-04-25

**Authors:** Charlotte Hommerberg, Anna W. Gustafsson, Anna Sandgren

**Affiliations:** 1 Department of languages, Linnaeus University, SE-35195 Växjö, Sweden; 2 Centre for Languages and Literature, Lund University, Lund, Box 201, SE-22100 Lund, Sweden; 3 Center for Collaborative Palliative Care, Department of Health and Caring Sciences, Linnaeus University, SE-35195 Växjö, Sweden

**Keywords:** Battle, Blogs, Burden, Cancer, Metaphors, Imprisonment, Journey, Palliative care, Patient narratives

## Abstract

**Background:**

The significance of metaphors for the experience of cancer has been the topic of extensive previous research, with “Battle” and “Journey” metaphors standing out as key. Adaptation to the patient’s use of metaphor is generally believed to be an important aspect of person-centered care, especially in palliative care. The aim of this study was to investigate the use of metaphors in blogs written in Swedish by people living with advanced cancer and explore possible patterns associated with individuals, age and gender.

**Methods:**

The study is based on a dataset totaling 2,602,479 words produced some time during the period 2007–2016 by 27 individuals diagnosed with advanced cancer. Both qualitative and quantitative procedures were used, and the findings are represented as raw frequencies as well as normalized frequencies per 10,000 words. Our general approach was exploratory and descriptive. The Mann-Whitney U test was used to analyze statistical significance.

**Results:**

Our results confirm the strong foothold of “Journey” and “Battle” metaphors. “Imprisonment” and “Burden” metaphors were also used by the majority of the individuals. The propensity to use metaphors when describing the cancer experience was found to differ extensively across the individuals. However, individuals were not found to opt for one conceptualization over the other but tended to draw on several different metaphor domains when conceptualizing their experience. Socio-demographic factors such as age or gender were not found to be strong predictors of metaphor choice in this limited study.

**Conclusions:**

Using a range of different metaphors allows individuals with advanced cancer to highlight different aspects of their experience. The presence of metaphors associated with “Journey”, “Battle”, “Imprisonment” and “Burden” across individuals could be explained by the fact that the bloggers are part of a culturally consistent cohort, despite variations in age, sex and cancer form. Awareness of metaphors commonly used by patients can enhance health professionals’ capacity to identify metaphorical patterns and develop a common language grounded in the patients’ own metaphor use, which is an important requisite for person-centered palliative care.

## Background

The past few decades have seen a considerable change in ideology, from a situation where patients with incurable conditions such as advanced cancer were often protected from awareness of their impending death [1] towards full disclosure and openness about the trajectory of the disease [2]. Having an open dialogue about serious and sensitive subjects such as approaching death can however be challenging to all parties involved [3].

There is some evidence that sensitive subjects, such as dying, can be more openly discussed using metaphors [4]. Metaphors are ways of understanding and describing abstract phenomena in terms of something that is more tangible. According to Conceptual Metaphor Theory, a highly influential model for metaphor research, metaphors are seen not only as consciously and creatively used phrases, but as a powerful cognitive phenomenon where our every-day use of language both reflects and affects our thinking in ways that often remain subconscious [5]. For instance, a word like *heavy* when used in the context of cancer illness associates the experience of living with the illness with physical weight. A similarity relation is thereby construed between the more concrete domain of meaning (labelled the source domain) and that which is abstract, i.e. the illness experience.

Metaphors are selective in the sense that they can highlight particular aspects of the illness experience and background others [6]. The research literature on metaphors and cancer has often focused on metaphors associated with the “Battle” and “Journey” domains [7–9], which are sometimes portrayed as opposite ways of conceptualizing the experience of the illness: “Battle” metaphors have been described as aggressive and masculine [8, 10, 11] and “Journey” metaphors as more peaceful, offering a reflective perspective [8, 12].

Attentiveness to the patient’s personal narrative [13], including the experiences and perceptions of the patient, is a central element of the caring process in a person-centered approach [14]. The use of metaphors is believed to be significant in the development of a common language which can enrich the relationship between the patient and health professional, as long as the patients’ own use of metaphors is allowed to guide the communication [10]. However, Southall’s [15] review of the existing literature on metaphors and palliative care shows that the focus of previous studies has often been health professionals’ use of metaphors, whereas there is a paucity of studies that actually highlight patients’ use.

The emergence of online fora has not only created important communicative spaces for people experiencing illness and family members wanting to discuss their experiences. Such narratives and discussions of illness can also provide a rich empirical resource for studies of authentic illness-related language use. The project Metaphor in end-of-life care (MELC) has offered a corpus-assisted analysis of online discussion forum posts written by people living with advanced cancer in the UK [6, 9]. Our study builds on the work carried out in this MELC project; however, we explore personal blogs, which is a slightly divergent genre involving more reflective writing and less interaction. To our knowledge, our study is the first one to offer a quantitative corpus analysis of online data in the palliative care context from another language than English.

The aim of this study was to investigate the use of metaphors in blogs written in Swedish by people living with advanced cancer and explore possible patterns associated with individuals, age and gender.

## Methods

### Design

The study combines computer-assisted qualitative and quantitative analyses of a dataset (corpus) totalling 2,602,479 words.

### Setting and sample

This study is based on data from blogs written in Swedish by individuals with advanced cancer. The blogs were identified by manually scanning the internet for relevant materials, using the broadest possible approach, such as key word searches, following links on cancer websites and forums and following links among the bloggers themselves. Only those blogs where the authors identified themselves as having advanced cancer were included for further scrutiny. A list of 42 blogs resulted from this initial search. Each blog was subsequently considered in relation to the following inclusion criteria, which are based on ethical reflections on the use of online data for research purposes: the blogger is a publically well-known figure and/or has explicitly stated s/he wants the blog to help others. All bloggers who were still alive were contacted to obtain consent.

In total, 27 blogs were included (median age 37), 21 of which were written by women (median age 36) and six by men (median age 44). Age refers to the point of the blogger’s death, or, for those still living, their age at the point in time when the blog was downloaded. The three most common cancer diagnoses were breast cancer (*n* = 9), colon cancer (*n* = 5) and gynaecological cancer (*n* = 4). The bloggers’ prior experience with public writing differs across the cohort, with three individuals identifying themselves as professional writers.

### Data collection

Several of the blogs had been initiated prior to the moment where the 27 individuals were informed that their cancer was incurable. To ensure maximum relevance in relation to our purposes, each blog was processed manually in search of the post where the information about the prognosis and/or the palliative care was introduced for the first time. The dataset was downloaded from this blog post and onwards, resulting in a corpus of 2,602,479 words, consisting of blog posts which had been uploaded during the period 2007–2016. The downloaded blogs differ in number of words (from 13,734 to 335,239) and extension in time (from a few months to more than 6 years). All quantitative data presented in this article are therefore normalized as instances per 10,000 words. The material is stored in a password-protected corpus managed by Språkbanken (the Swedish Language Bank, Gothenburg University).

### Data analysis

This study is grounded in Conceptual Metaphor Theory [5] as theoretical framework and offers a linguistic approach using corpus methods. Corpus techniques enable large collections of text data to be systematically investigated regarding potential patterns in metaphor use [16]. Our research procedure followed the main steps of the UK-based project Metaphor in end-of-life care [6]. The data analysis included the following steps:

*Step 1.* Processing of a pilot material consisting of two different types of data from three different groups: blogs written by patients and family members and interviews with patients, family members and health professionals carried out within the research programme Dignity in palliative care at The Center for Collaborative Palliative Care, Sweden. A selection of 90,000 words from this pilot material was processed manually, noting every instance of a metaphorically used word or phrase that was used to describe some aspect of the cancer experience. A Swedish dictionary [17] was used as a reference tool to determine basic word meanings. At this stage, the data were approached from an open-ended perspective, without preconceived ideas based for instance on the research literature, using a modified version of the well-established linguistic Metaphor Identification Procedure [18] according to which all expressions that are used in their non-literal sense are seen as potential instances of metaphor.

*Step 2.* Grouping of the words and phrases that emerged from Step 1 under tentative metaphoric source domain labels. Four source domains, “Journey”, “Battle”, “Imprisonment” and “Burden”, stood out as more productive and systematic than the rest in terms of the range of different words that were associated with them. Other potential source domains were identified, for instance “Building” and “Weather”, but these were less salient. Based on these preparatory steps, a decision was taken to explore the use of metaphors associated with “Journey”, “Battle”, “Imprisonment” and “Burden” in the entire dataset.

*Step 3.* Processing of the entire dataset of patient blogs. Computer-assisted (Språkbanken, KORP) lemma searches of the entire dataset using the wordlists established in Steps 1 and 2 as initial search words and, in accumulative fashion, adding new expressions that were used metaphorically in the co-text of these search words. The accumulation of words was deemed exhaustive at the point where no more metaphorical expressions were found, resulting in a list of 170 word types for all the four source domains combined. Our analysis of the 27 blogs is based on lemma searches using these 170 expressions as search words, for each instance determining whether the expression was used metaphorically in an illness-related context and not in its literal sense or metaphorically in a different context. The data were divided between and processed by two analysts with linguistics as disciplinary background and equal familiarity with the material. Elusive instances were continuously scrutinized in critical discussions.

*Step 4.* Examination of the distribution of metaphorical expressions across individuals and groups, using sociodemographic information disclosed by bloggers as a means to explore potential patterns associated with the bloggers’ age and gender. The Mann-Whitney U test was used to analyze whether any differences between groups were statistically significant.

All examples presented in this article are retrieved from the Swedish language dataset and translated into English with as little rewording as possible. This means that quotations are not searchable, which is a further means of protecting the bloggers’ identities. It should be made clear that the semantics of words and phrases is never completely identical in different languages and the translations are therefore to be seen as approximations of the original Swedish word meanings. Examples are labelled with codes offering sociodemographic information disclosed by the blogger (W = woman, M = man, number = age, lower case letters a and b are used to distinguish bloggers of the same sex and age).

## Results

### “Journey”, “Battle”, “Imprisonment” and “Burden”

This section presents the results of the processing of the entire dataset as described in step 3 for metaphorical words and expressions associated with the four source domains, “Journey”, “Battle”, “Imprisonment” and “Burden”. A total of 7286 instances of metaphor use associated with these four categories were identified in step 3. Table 1 displays the distribution of word types across source domains and the total frequency of metaphorical uses of these word types added together for each source domain.

**Table 1:** Total instances of metaphors and relative distribution

Source domain	Word types *N* = 170	Instances and relative distribution *N* = 7286
Journey	61	3597 (49%)
Battle	61	2587 (36%)
Imprisonment	30	594 (8%)
Burden	18	508 (7%)

The number of word types gives an indication of the relative productivity and systematicity of these four source domains. “Journey” and “Battle” metaphors occupied a fronted niche in the dataset as a whole, as regards both number of word types and frequency of metaphoric use, while expressions associated with “Imprisonment” and “Burden” were considerably fewer, as regards both word types and frequency.

### Journey

Journey metaphors were most common in the data. The most frequent metaphorically used expressions were ‘road’ (*väg*), ‘come back’ (*komma tillbaka*), ‘turn’ (*vända*), ‘go through’ (*gå igenom*) and ‘goal’ (*mål*). The framing of the illness experience in terms of a journey or movement in spatial terms was present in the patients’ narratives in both single-word expressions that occur on their own and more complex extended metaphorical uses. In the following excerpt, for instance, the idea that having advanced cancer is like being on a journey is clearly present in a creative utterance including several expressions that draw on the domain of Journey:

*I want to climb off but there is no stop button. No way of making the carriage stop so I just have to ride along.* (W28).

The idea of movement forward in spatial terms is also present in isolated expressions, as in the following example where the cancer treatment is described as something that the patient has *gone through*:

*Most people who have coped with and gone through cancer and chemotherapy say that it’s the worst thing they have ever experienced.* (M35).

### Battle

Words with vague or general meanings, such as ‘fight’/‘struggle’ (*kämpa*, verb), ‘hit’/‘strike’ (*drabba*), ‘fight’/‘battle’/‘struggle’ (*kamp*, noun), ‘give up’ (*ge upp*) and ‘beat’/‘strike’/‘hit’ (*slå*), were among the most frequent Battle-related words. Explicitly war-related expressions, such as ‘enemy’ (*fiende*), ‘war’ (*krig*) or ‘combat’ (*strid*), were not as common. Battle metaphors were used in both complex, extended ways and in utterances where only a single word was Battle-related. These two variants are exemplified in the following excerpts.

*I will spit in my palm and roll up my sleeves. I will fight to the last drop of blood.* (W48).

*To be honest, I have in a way already given up. I find myself hedging and reminding myself that I won’t be cured. This may upset some people.* (W58).

### Imprisonment

The most frequent metaphorical expressions associated with the source domain of Imprisonment were ‘let go’ (*släppa*), ‘free’ (*fri*), ‘stuck’ (*fast*), ‘delimit’ (*begränsa*), ‘freedom’ (*frihet*) and ‘get stuck’ (*fastna*). The excerpt below offers an elaborate use of metaphors involving several expressions that are associated with Imprisonment.

*It is as if someone had put a free bird in a cage. Taken away its freedom and left if without alternative to affect its destiny.* (W28).

Expressions associated with Imprisonment were also present as single words as in the following example where physical symptoms related to the treatment are portrayed as delimiting the person.

*This is not how my body should be, I shouldn’t be delimited like this, that this is the price I pay to stay alive just has to be accepted unconditionally*. (M47).

### Burden

The most frequently used metaphorical expressions associated with the domain of Burden were ‘heavy’ (*tung*), ‘carry’ (*bära*), ‘load’ (*belastning*), ‘lift’ (*lyfta*) and ‘weight’ (*tyngd*). This excerpt offers an elaborated example of Burden metaphors, involving several expressions that are associated with this domain:

*I’ve been carrying this burden for four years now. Always this backpack…* (W58).

The following example illustrates use of a single Burden-related word:

*To have a relapse during ongoing treatment is heavy.* (W33).

### Combined use of Journey, Battle, Imprisonment and Burden

In this study, we have grouped the words used by the bloggers according to source domain. However, the patients’ utterances often involve references to more than one source domain at the same time. The following example employs expressions associated with both Journey and Battle, suggesting that these two conceptualizations of the illness are simultaneously accessible to the blogger:

*After everything I’ve gone through I still stand firmly with my fighting spirit and I will not give up.* (W28).

In a similar vein, the example below uses expressions associated with both Journey and Burden:

*You who follow me on this journey are many. That makes me happy and the journey somewhat less heavy to make.* (W35b).

Furthermore, the idea of being caught by the illness, being in its grip, i.e. imprisoned, is present in the following utterance which also involves Battle-related expressions.

*…my own fear of seeing more people suffer, suffer in the grip of the illness and lose the battle they have been fighting so hard and for so long.* (M47).

### Comparison across individuals and between age and gender groupings

**Fig. 1:**
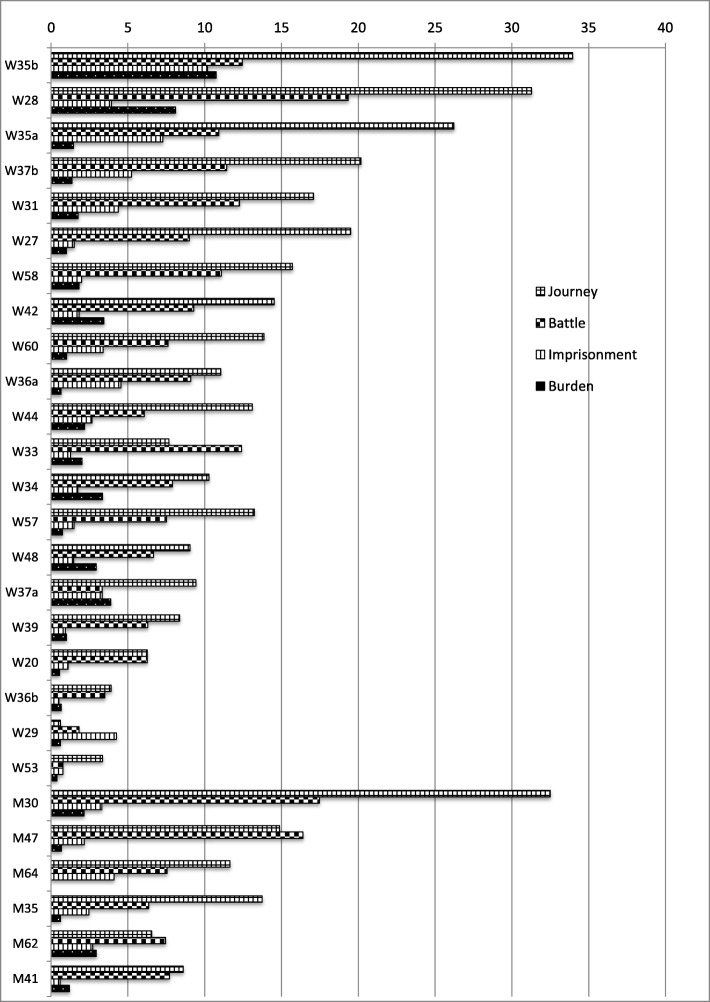
Individuals’ use of Journey, Battle, Imprisonment and Burden metaphors

The total use of metaphorical expressions associated with all of the four source domains, Journey, Battle, Imprisonment and Burden, differs extensively across the 27 bloggers, in total from 67 instances per 10,000 words to 5 instances per 10,000 words. Figure 1 shows the distribution of individual bloggers’ use of metaphors.

Figure 1 shows that expressions invoking some form of journey or spatial movement related to the cancer experience were used by all of the bloggers. Journey metaphors were the most common types of metaphor among all but five bloggers. As exceptions to the more general trend, the texts written by W33, M47 and M62 were dominated by Battle-related metaphors, and W20’s text had an equal proportion of Journey and Battle metaphors. Only one blog, W29, displayed a dominance of Imprisonment metaphors. We also note that one individual, M64, deviated from the trend in the sense that no Burden metaphors were identified in this blog based on the 18 word types that were used as search words.

**Fig. 2:**
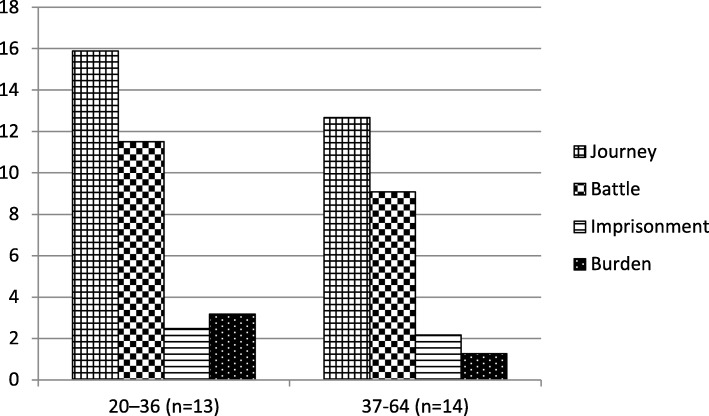
The proportions of use of metaphors per age group (instances per 10,000 words)

The patterns in the bloggers’ use of metaphors based on their age are presented in Fig. 2. The figure divides the bloggers into two groups, based on the median age. The data from the two age groups amount to 932,473 words for the younger bloggers and 1,670,006 words for the older bloggers.

As shown by Fig. 2, the relation between the metaphor categories remained more or less equal across the two age groups (NS).

**Fig. 3:**
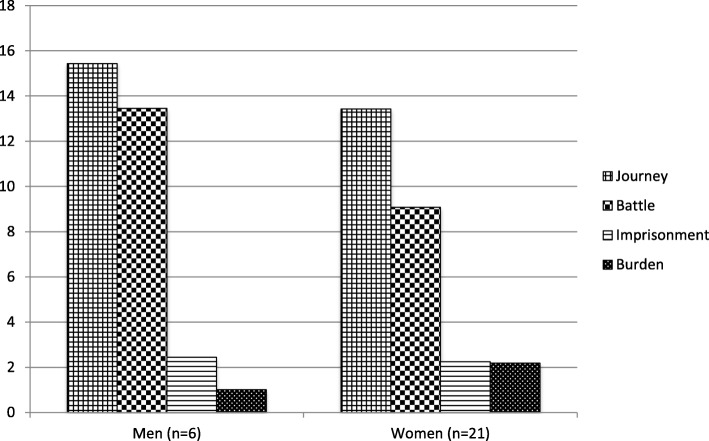
The proportions of use of metaphors per gender group (instances per 10,000 words)

The blog material produced by the women (2,086,546 words) amounted to approximately four times more than the data produced by the men (515,933 words). Fig. 3 shows normalized frequencies for these two groups’ use of metaphors associated with the four source domains examined here.

Journey metaphors dominated both women’s and men’s blogs and were used to an equal proportion by both groups. The slight divergence in the use of Battle metaphors was not statistically significant. Imprisonment and Burden metaphors were the least frequent for both groups.

## Discussion

In this study we have investigated the use of metaphors in a dataset consisting of blogs written in Swedish by people living with advanced cancer and observed possible patterns associated with individuals as well as age and gender groupings. Metaphors from the Journey and Battle domains occupied an uncontested niche in our data as a whole, making up 85% of the metaphors associated with the four source domains explored here. Two other metaphor domains that have received less attention in the research literature, Imprisonment and Burden, occurred less frequently but were still found to be relatively productive sources of metaphorical meaning.

The comparison across the bloggers showed that individuals did not opt for one conceptualization over the other but tended to draw on several different ways of conceptualizing their experience by means of metaphors. The need to use a range of metaphors may be indicative of the contradictory and conflicting experiences that people with advanced cancer have. Due to the effects of the illness and the emotional stress caused by their situation, they may no longer be able to engage in and profit from their ordinary life in the same way as before, leading to a feeling of both loss of control, having their lives put on hold [19] and a need to confront their forthcoming death in different ways [20]. Using metaphors associated with all the four source domains explored here can be seen as a way of articulating these different perceptions and physical as well as emotional experiences.

Our results confirm the strong foothold of Journey and Battle metaphors which has been noted in previous studies of a large English-language dataset of online discussion forum posts [6, 9] as well as in a limited dataset of Spanish-language blogs [21]. The high frequency of metaphors related to the domain of Journey, both in creative, complex metaphorical utterances and in more conventionalized uses, is indicative of the widespread significance of this metaphorical construct. The Journey metaphor has been seen as a helpful, constructive metaphor for patients with cancer [12]. According to Reisfield and Wilson [8] the Journey metaphor offers positive elements such as ‘new sources of meaning’, ‘opportunities for personal growth’ and ‘a vision of a deeper meaning of life’. However, Semino et al. [6, 9] observed that patients’ use of Journey metaphors is not necessarily empowering, because they can express a sense of passivity or isolation.

Furthermore, more than one third of the instances found in our study were associated with the domain of Battle in some form, and this is therefore a source of metaphorical meaning that had an uncontested significance for the bloggers in this study. Battle metaphors have received a great deal of attention in the research literature [e.g. [6–10, 15, 21–25]]. They have often been described as emphasizing power, strength, agressivity and masculinity [8, 10, 11]. Battle metaphors can function as conceptual resources to counteract feelings of power loss or helplessness that the illness gives rise to. But this conceptual construct can also cause feelings of failure and guilt, because it can position a patient referred to palliative care as someone who failed the treatment rather than someone who is failed by the treatment [8]. Semino et al. [9] as well as Gustafsson & Hommerberg [24] demonstrate that battle metaphors can be used to invoke both empowering and disempowering scenarios. Battle metaphors are generally absent from policy documents as well as self-help books and information materials for patients in the UK [26], Australia [27] and Sweden [28], conceivably because of the negative implications that they may have. Yet they are ubiquitous in medical discourse [22], the media [29, 30] and cancer campaigns [31] and, as demonstrated by our results, often used by people blogging about their experience of living with advanced cancer, to express different ways of coping [32].

Our results do not show any strong indication that choices in metaphor use should be associated with age. The general trend is that metaphor use seems at large to be consistent across the two age groups, which in itself is a noteworthy result. Further studies would be needed in order to explore possible correlations between age and metaphor use in the context of cancer illness. Furthermore, our comparison of men’s and women’s metaphor use shows that these two groups adopt metaphors more or less equally from the source domains explored here. This is interesting given that Battle metaphors have previously been described as associated with masculinity [8, 10] and studies in psychology and palliative cancer care have indicated that men and women have different communication styles [33]. One limitation of our study is the imbalance between male and female bloggers in the dataset, which conceivably reflects a real-world situation where female bloggers are more inclined to communicate online about their experiences. Further studies of mens’ and women’s use of metaphors would be needed in order to shed conclusive light on potential gender differences.

It has been argued that the use of metaphors may improve communication with patients [34] and that physicians should tailor their use of metaphorical language to suit the individual patient’s characteristics [8]. For this purpose, health professionals need to be able to listen actively to patients’ own use of metaphors [10]. Insights into how people living with advanced cancer use metaphors in authentic settings which are not immediately connected to the health care environment can improve the requisites for a common language that enriches the relationship between the patient and health professionals. Enhanced awareness of what is culturally typical can also help health professionals become more conscious of their own use of metaphors so that they do not introduce metaphorical conceptualizations unknowingly [25]. Southall [15] describes the health science literature as overwhelmingly positive regarding the function of metaphor in palliative care, but calls for enhanced awareness of the potential risk since “[a] metaphor which might bring solace to one person might be a marker of terror for another” (p. 312).

Our results showed remarkable consistency across the bloggers in the sense that they tended to adopt metaphors from all four of the source domains that were investigated in this study. This also concerned the less visible domains of Imprisonment and Burden. This consistency could be explained by the fact that the bloggers were part of a culturally consistent cohort, despite variations in gender, age, cancer form and blog style. This finding is in line with previous observations in medical anthropology, where it has been noted that people’s ways of coping with and talking about illnesses and experiences of physical or emotional pain using metaphors are related to culture [35, 36], and studies of metaphor in general have also confirmed the influence of culture [37]. Since our results agreed with previous corpus studies of patients’ use of metaphors in English language data [6, 9], it can be suggested that the British and Swedish cultures are fairly similar in their entrenched conceptualizations of cancer. It is particularly noteworthy that these similarities persisted despite the fact that our study used a dataset of personal blogs, which is a slightly different and less interactive genre than the discussion forums that were analyzed in the British study. The metaphoric source domains that we have explored in this study are not only accessible to the bloggers at different points in their narratives but can be simultaneously accessible to draw on as resources when describing and attempting to cope with their often overwhelming and contradictory experiences of life with advanced cancer [32]. Such combinations of metaphor domains were also observed by Semino et al. [6] to occur in English language data.

This study was based on texts written by people who have chosen to share their experiences of advanced cancer in open blogs online. This means that the data were produced by a self-selected group which might not be representative of the whole population. The fact that the materials were produced by a diversified group who have chosen to write about their life with advanced cancer in rather different ways can nonetheless be seen as an asset and could be taken to indicate more general significance of the results; despite variations in blog style, the same metaphor domains were used as sources when describing the illness experience. It should be acknowledged that due to our desire to offer a quantitative representation of the entire dataset, this study does not aspire to reflect the complex functions of the different metaphor domains that have been studied, but only focuses on their presence in the dataset. A qualitative analysis of the function of the metaphors in relation to coping strategies is offered in another article [32].

## Conclusion

The propensity to use metaphors when describing the cancer experience differed extensively across the bloggers in this study. However, there was remarkable consistency across the bloggers in that they tended to use expressions drawing on all of the four source domains investigated here, Journey, Battle, Imprisonment and Burden, to describe facets of the cancer experience. We therefore cannot conclude that certain individuals favour only certain metaphors when conceptualizing their illness. Instead, they seem to need several different metaphors to verbalize their experiences, emotions and perceptions. Health professionals need to be able to listen actively to such richness and variation in patients’ own use of metaphors in order to let the patients’ articulation of their experience guide the communication. Further studies involving more individuals would be needed in order to confirm tendencies in usage across age and gender groupings. Another angle that remains to be explored is to what extent individuals’ metaphor use changes during the progression of the illness. In addition, future studies could explore how people living with advanced non-cancer diagnoses use metaphors.

## Abbreviations

NS: Not statistically significant
